# Toward Asymmetric Synthesis of Pentaorganosilicates

**DOI:** 10.1007/s11244-018-0967-5

**Published:** 2018-04-20

**Authors:** Leon J. P. van der Boon, Shin-ichi Fuku-en, J. Chris Slootweg, Koop Lammertsma, Andreas W. Ehlers

**Affiliations:** 1 Department of Chemistry and Pharmaceutical Sciences, Faculty of Sciences, Vrije Universiteit Amsterdam, De Boelelaan 1083, 1081 HV Amsterdam, The Netherlands; 2 Department of Chemistry, Graduate School of Science, Hiroshima University, 1-3-1 Kagamiyama, Higashi-Hiroshima, 739-8526 Japan; 3 Present Address: Van ‘t Hof Institute for Molecular Sciences, University of Amsterdam, Science Park 904, 1098 HX Amsterdam, The Netherlands; 4 Department of Chemistry, University of Johannesburg, Auckland Park, Johannesburg, 2006 South Africa

**Keywords:** Asymmetric catalysis, Silicates, Spirosilanes

## Abstract

Introducing chiral silicon centers was explored for the asymmetric Rh-catalyzed cyclization of dihydrosilanes to enantiomerically enriched spirosilanes as targets to enable access to enantiostable pentacoordinate silicates. The steric rigidity required in such systems demands the presence of two naphthyl or benzo[b]thiophene groups. The synthetic approach to the expanded spirosilanes extends Takai’s method (Kuninobu et al. in Angew Chem Int Ed 52(5):1520–1522, 2013) for the synthesis of spirosilabifluorenes in which both a Si–H and a C–H bond of a dihydrosilane are activated by a rhodium catalyst. The expanded dihydrosilanes were obtained from halogenated aromatic precursors. Their asymmetric cyclization to the spirosilanes were conducted with [Rh(cod)Cl]_2_ in the presence of the chiral bidentate phosphane ligands (R)-BINAP, (R)-MeO-BIPHEP, and (R)-SEGPHOS, including derivatives with P-(3,5-t-Bu-4-MeO)-phenyl (DTBM) groups. The highest enantiomeric excess of 84% was obtained for 11,11′-spirobi[benzo[b]-naphtho[2,1-d]silole] with the DTBM-SEGPHOS ligand.

## Introduction

Chirality is a cornerstone in chemistry, crucial to live, and increasingly important to materials science. The synthesis of chiral organic molecules has become commonplace [1]. Simple examples of the numerous ones available are Knowles’s synthesis of l-DOPA and Sharpless’ asymmetric epoxidation resulting in compounds of high enantiomeric excess (*ee*) [2, 3]. Common approaches to introduce chirality are nucleophilic substitution of pro-chiral systems and asymmetric catalysis [4]. Once a chiral carbon center is formed, it retains its chirality in the absence of competing associative/dissociative reactions.

In strong contrast to compounds having chiral carbon centers, those with a chiral silicon center are not found in nature. Kipping reported the first optically active silicon compounds early in the previous century [5], and the first examples of chiral resolution on a practical scale were presented much later [6]. Extensive pioneering work on stereospecific reactions towards chiral silicon compounds was done by the group of Sommer [7]. During the same period, Corriu and coworkers laid the foundation of much that we know today about stereoselectivity in substitution reactions on silicon [8]. It is evident that chiral silicon compounds can only be acquired synthetically, some of the more recent methods towards these compounds are excellently reviewed elsewhere [9–11].

Chirality in tetracoordinate silicon derivatives has become a topic of recent interest and is driven by asymmetric catalysis using rhodium complexes carrying chiral diphosphane or diene ligands [12]. Examples include chiral silicon-ferrocenes by enantioselective activation of C–H bonds, enantioselective 1,4-silicon shifts from aryl to alkyl groups, and the chiral silylation using iridium complexes [13–15]. Here, we describe our synthetic approach toward chiral silanes for which we have a need in our program on chiral pentacoordinate silicon compounds.

It is important to recognize that the synthesis of enantiostable tetracoordinate silicon compounds is inherently more challenging than their carbon counterparts due to the tendency to form pentacoordinate intermediates or products, which readily undergo racemization [16]. The bigger atomic radius of silicon stabilizes such species, termed silicates, whereas the pentacoordinate carbon ones are instead transition structures [17]. Before proceeding with our synthesis on tetracoordinate silicon compounds, it is relevant to highlight the potential involvement and characteristics of silicates.

Pentacoordinate Si-intermediates are well documented for S_N_2 reactions. Racemization at the Si-center occurs readily via Berry pseudorotations (BPR),[18–20] causing isomerization between different trigonal bipyramid conformations via a low energy square pyramidal transition state. This change of geometry underlies the racemization process and can only be inhibited by increasing the barriers for isomerization. Recently, Strohman’s group provided such an example of selective inhibition for an S_N_2-type substitution at a prochiral silicon center [21]. They were able to obtain a chiral compound by selectively hampering one of the BPRs of the five coordinate Si-intermediate.

**Fig. 1:**
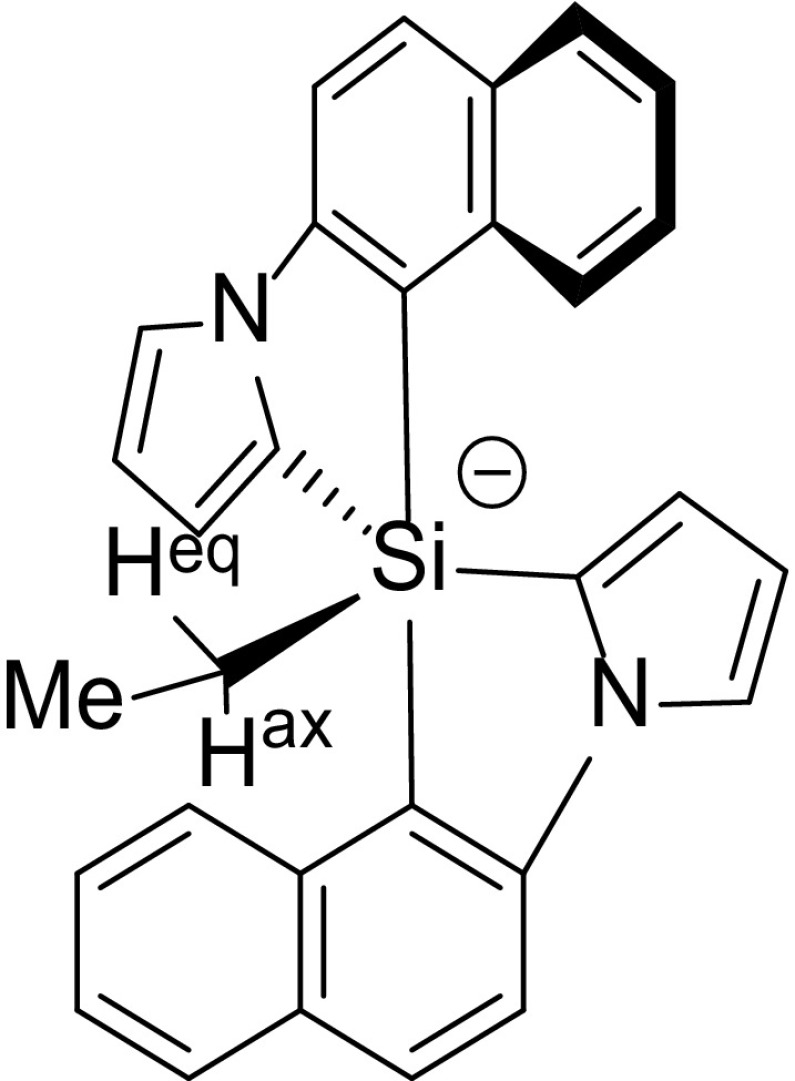
Silicate with diastereotopic protons

Earlier, we have shown computationally how to impede Berry pseudorotations to obtain chiral, enantiostable pentaorganosilicate salts and presented a synthetic method to accomplish this [22]. Silicate anions with five carbon substituents are usually short-lived species, observable only by mass spectrometry or low temperature NMR, but their stabilization increases substantially when four of the five Si–C bonds are formed by two bidentate ligands, such as biphenyls [23–25]. Such stable pentaorganosilicates, but with larger aromatic ligands, prevent formation of a square pyramidal conformation, thus hampering the BPR and preventing racemization. NMR spectroscopy revealed for ethyl,bis([2] naphthylpyrrol)silicate (Fig. 1) a BPR barrier of over 21 kcal mol^− 1^ and showed two diastereotopic ethylene hydrogens [22]. However, demonstrating unequivocally the inhibition of BPRs also demands an enantiomerically pure silicate to be isolable. It is in this context that we pursue the synthesis of chiral silanes, because these are the starting point to generate silicates. Therefore, we seek an enantioselective synthetic approach to symmetrical spirosilanes with large aromatic groups.

**Scheme 1:**
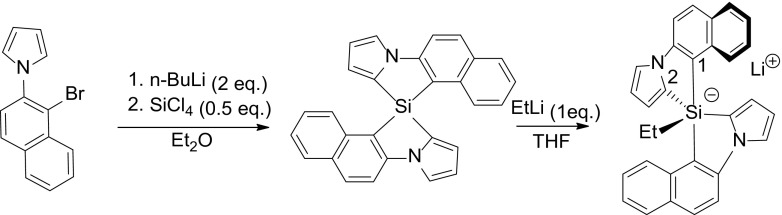
Lithiation route towards racemic pentaorgano-silicates

**Scheme 2:**
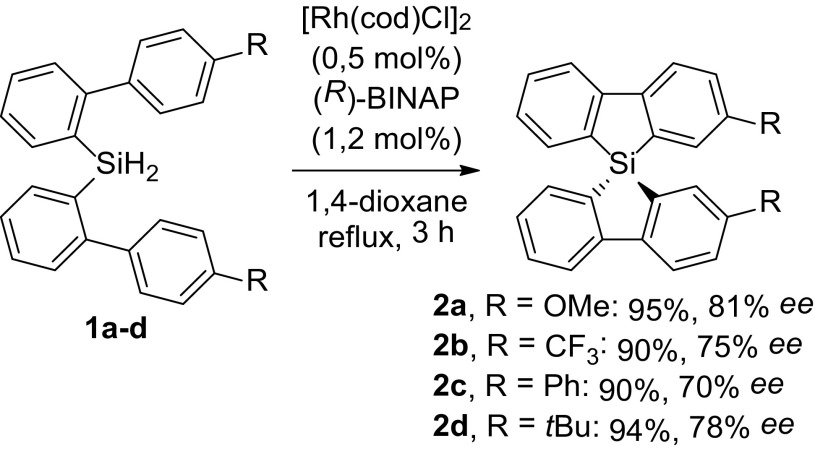
Asymmetric rhodium catalysed route towards various spirosilanes

Conventionally, spirosilanes are obtained from the reaction of SiCl_4_ with a dilithiated biaryl system generated from its dihalide (Scheme 1) [22]. Obtaining the chiral products than requires separation into the enantiomers by, e.g., chiral HPLC [23]. We envisioned that it might be advantageous to explore an enantioselective synthesis. Our approach is based on recent work by Takai and coworkers, who (a) reported on the synthesis of 9H-9sila-fluorenes using a rhodium catalyst for Si–H and C–H bond activation [26] and (b) showed that spirosilanes could be obtained in high enantiomeric excess from dihydrosilanes on using a Rh-catalyst with a chiral (R)-BINAP diphosphane ligand (Scheme 2) [27]. The mechanism proposed for the reaction involves sequential oxidative additions of the Rh-catalyst at an aromatic C–H bond and a Si–H bond, followed by H_2_-elimination and reductive elimination to a monohydrosilane, which undergoes the same catalytic cycle to give the spirosilane [28].

**Scheme 3:**
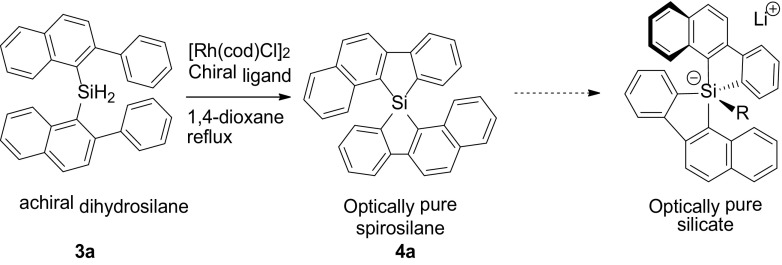
Asymmetric route towards optically pure pentaorgano-silicates

We sought to expand the scope of the chiral synthesis by using sterically more demanding substituents than the biphenyl group to obtain in high enantiomeric excess ‘bisligated’ spirosilanes carrying naphthalene or benzo[b]thiophene groups (Scheme 3). Such systems would ideally confirm the ability to control the chirality of silicates and potentially reduce racemization of chiral silanes by the contaminating presences of silicates. At the same time, we realized that the increased steric bulk might affect the cyclization reaction unfavorably.

**Scheme 4:**
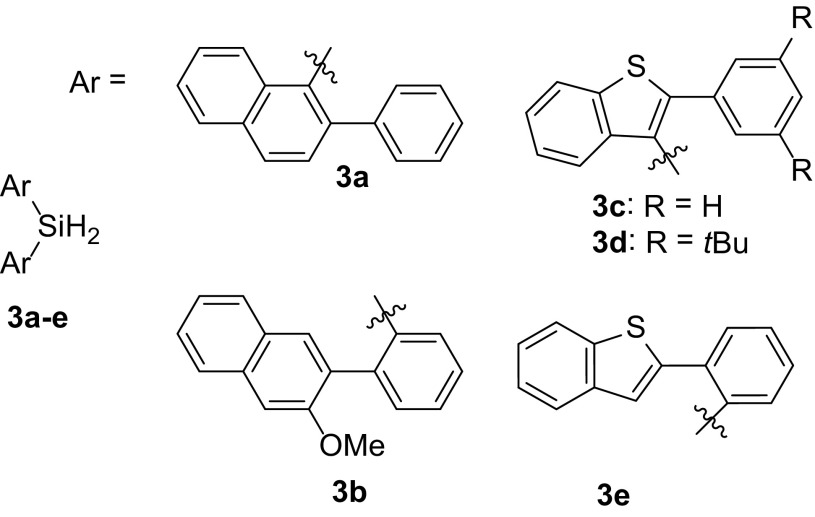
Dihydrosilanes containing bulky ring systems

Derivatives of the larger aromatic substituents 2-phenylnaphthalene and 2-phenylbenzo[b]thiophene that were examined in this study are shown in Scheme 4. The selection of the two aromatic cores (naphthalene and benzo[b]thiophene) was derived from silicates that have been shown to be configurationally rigid. Double ring closure of dihydrosilanes **3a** with two 2-phenylnaphthyl groups and **3c** with two 2-phenylbenzothio[b]thiophenyl groups will result into the two targeted spirosilanes. Additional t-butyl groups increase the sterics of **3d**. Dihydrosilanes **3b** and **3e** were selected to resemble more the noted examples where the silicon atom is substituted on the phenyl ring. The methoxy-group on **3b** would also hinder unwanted reactions of the rhodium catalyst with the 3-CH bond of the naphthalene ring.

## Results and Discussion

To explore the increased size of the substituents on the synthesis of dihydrosilanes and the subsequent enantioselective cyclization to spirosilanes by using the procedure of Takai and coworkers [23], we felt it important to be able to reproduce their result as benchmark.

**Scheme 5:**
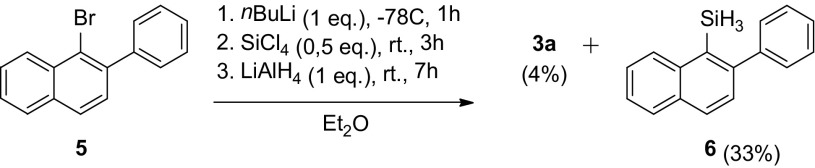
Synthetic route towards naphthalene-phenhyl di- and tri hydrosilane

The synthesis of the dihydrosilanes with larger aromatic groups proved to be more difficult than those with the biphenyl groups and could only be accomplished by introducing a silyl group on the larger rings, i.e., the naphthalene and benzo[b]thiophene groups. The most productive synthetic approach was to lithiate the bromo precursors with either *t-*BuLi or *i*Pr-MgCl·LiCl, followed by treatment with SiCl_4_ to obtain the dichlorosilane and treatment with LiAlH_4_ to form the dihydrosilanes, which could only be obtained in poor yields. Illustrative is the synthesis of silane **3a** from 1-bromo-2-phenylnapthalene with a yield of only 4% with trihydrosilane **6** as major isolated product in 33% yield (Scheme 5). Evidently, steric congestion plays a significant role and hampers the second nucleophilic addition at SiCl_4_. The naphthyl crowding effect is even more pronounced on introducing a silyl group at the phenyl group, as in **3b**, which we were unable to obtain.

**Scheme 6:**
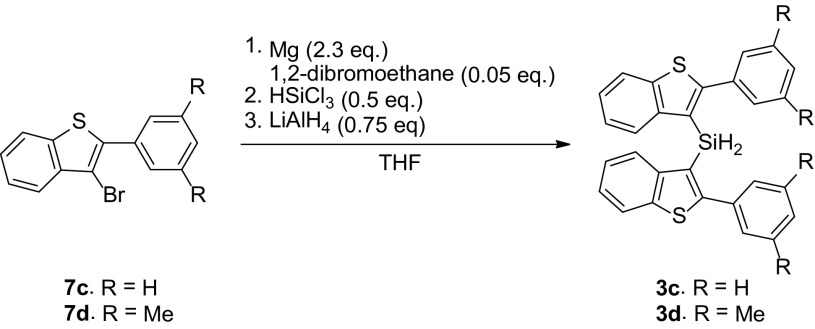
Grignard route towards benzothiophene dihydrosilanes

For 2-phenylbenzo[b]thiophene the synthetic access proved to be more favorable than for 2-phenylnaphthalene. (Scheme 6). Thus, **3c** could be prepared in 53% yield by reacting the Grignard reagent of bromide **7c** with subsequently HSiCl_3_ and LiAlH_4_. The product showed the expected Si–H interaction using ^1^H-NMR spectroscopy. Steric factors do have an influence on the accessibility of the benzo-[b]thiophene group, which became evident on introducing two *t-*Bu in the meta positions of the phenyl substituent as the synthetic procedure only resulted in replacement of the bromide of **7** for a hydrogen atom.

**Scheme 7:**
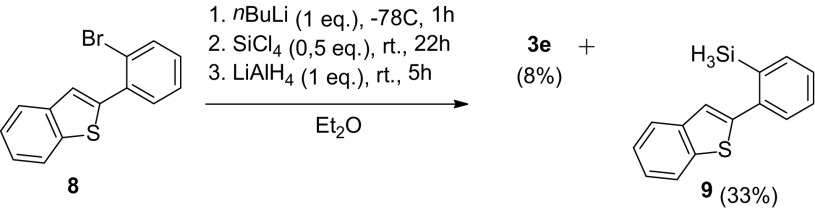
Lithiation route towards phenyl-benzothiophene di- and trihydrosilanes

In contrast to 2-phenylnaphthalene access silylation of the phenyl substituent ring of 2-phenylbenzo[b]thiophene (**8**) proved feasible, giving **3e**, be it in only 8% of impure material, besides trihydrosilane **9** as major product (Scheme 7). This again illustrates that steric congestion hampers the second nucleophilic addition at silicon.

### Asymmetric Catalysis

To assess the rhodium catalyzed asymmetric synthesis of dihydrosilanes, we re-examined the intramolecular cyclization of dihydrosilane **1a** using (*R*)-BINAP and [Rh(cod)Cl]_2_ to obtain the 2,2′-dimethoxy-9,9′-spiro-9-silabifluorene **2a** in 84% yield and 79% *ee* (Table 1), which is in excellent agreement with the reported 81% ee. We note that lower ee’s were obtained if the reaction was not executed under inert conditions and degassed solvents.

**Table 1:**
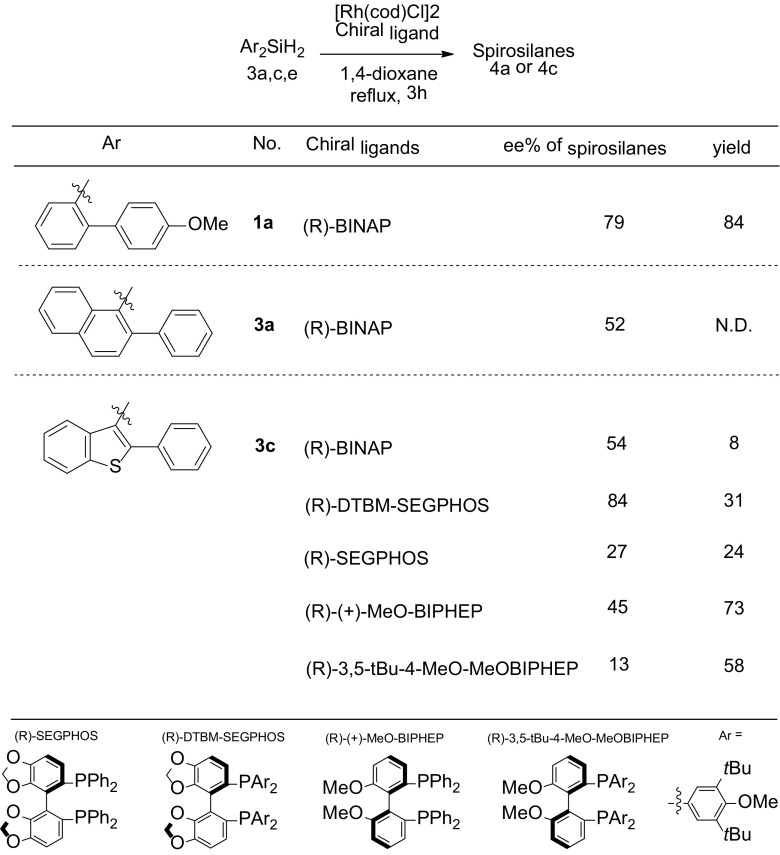
Screening of asymmetric route towards chiral sterically encumbered spirosilanes

The intramolecular double cyclization of the bulkier dihydrosilanes **3a** and **3c** using (*R*)-BINAP and [Rh(cod)Cl]_2_ yielded the corresponding spirosilanes **4a** (< 10%) and **4c** (8.1%) in 52 and 54% *ee*, respectively. Whereas we had expected higher *ee*’s, anticipating that steric congesting would positively influence the distinction between the formation of both enantiomers [29], the *ee*’s were, in fact, lower than that obtained for **2a**. This effect must be due to the larger ring substituents, which apparently reduces the enantioselectivity. Also the fact that minor impurities were obtained, which could be removed by washing with *n*-pentane to significantly reduce the yields (e.g., to 8.1% for **4c**), is also indicative for the steric sensitivity for cyclization.

In searching to improve the enantioselectivity, we explored other chiral ligands for the Rh-catalyzed bicyclization of **3c** (Table 1). Formation of spirosilane **4c** from **3c** using the chiral methoxy-phenyl ligand MeO-BIPHEP and the bulkier 3,5-*t-*Bu-4-MeO-MeO-BIPHEP gave still lower *ee*’s of 45% and even 13%, respectively. Apparently, the bulkier phenyl rings around the coordinating phosphorus ligand reduce the enantioselectivity of the reaction. It is than most intriguing that we found a much higher *ee* of 84% on using the quite large DTBM-SEGPHOS ligand, albeit with an expected lower yield of 31%. Clearly, with this bulky chiral ligand the transfer of chirality is effectively transferred in the Rh-catalyzed cyclization reaction. On removing the tBu and OMe substituents of the ligand’s two P-phenyl groups, i.e., SEGPHOS, the steric bulk of the ligand reduces and so does the *ee* to 27%, while also reducing the yield (to 24%) Evidently, the bidentate ligand systems BIPHEP and SEGPHOS have significant effects on the enantioselective cyclization, whereas they merely differ in carrying OMe versus 1,3-dioxole groups.

## Conclusion

This research has expanded the scope of the asymmetric Rh-catalyzed cyclization of dihydrosilanes to spirosilanes with more bulky ring systems. The successful synthesis of the naphthalene and benzo[b]thiophene dihydrosilanes **3a** and **3c** and their conversion to the corresponding spirosilanes **4a** and **4c** showed that the Rh-catalyzed cyclization can introduce modest to high enantioselectivity. The P-ligand systems that were used in the investigation range from (R)-BINAP to (R)-MeO-BIPHEP to (R)-SEGPHOS and include P-phenyl and P-(3,5-t-Bu-4-MeO)-phenyl groups. The highest *ee* of 84% was obtained for the formation of **4c** on using the Rh-ligand DTBM-SEGPHOS, with a modest yield of 31%. This finding shows that congested spirosilanes can be synthesized with modest to high enantioselectivity, which bodes well for the direct synthesis of enantiostable silicates. Our survey also reveals limitations such as the synthetic access of the required dihydrosilane precursors where the increased steric congestion has a debilitating effect, inhibiting the second nucleophilic substitution at silicon, thereby rendering trihydrosilanes; formation of byproducts also hampers product purification.

## Experimental Section

**General** All reagents were bought from Sigma–Aldrich and used as received unless specified otherwise. Pyridine was dried using activated molecular sieves (3 Å). Tetrahydrofuran (THF) was distilled subsequently from LiAlH_4_ and sodium/potassium alloy and diethyl ether from sodium/potassium alloy. *n*-Butyllithium was purchased as 1.6 M solutions in hexanes. Tetrachlorosilane was distilled and refluxed before use to remove HCl. Syntheses were performed using standard Schlenk techniques. NMR spectra were recorded on a Bruker Avance 400 (^1^H, ^13^C, ^29^Si, 2D spectra). NMR chemical shifts are internally referenced to the solvent for ^1^H (CHCl_3_: 7.26, THF: 3.58, CH_2_Cl_2_: 5.32) and ^13^C (CHCl_3_: 77.16, THF: 67.58, CH_2_Cl_2_: 53.84), and externally for ^29^Si to TMS. Melting points were measured on samples in sealed capillaries and are uncorrected. HR-ESI-MS measurements of silicates were measured on a Varian IonSpec FT-ICR mass spectrometer, for LR-MS a Bruker micrOTF system was used. Separation of enantiomers was performed on a Chiralpak IA column.

### Synthesis of 2-Bromo-4′-methoxy-biphenyl 10 [27]

 *t*-BuLi (1.6 M in pentane, 4.0 mL, 6.4 mmol, 2.1 eq.) was added to a solution of *p*-methoxyiodobenzene (936 mg, 4.0 mmol, 1.3 eq.) in THF (5.0 mL) at − 78 °C. After 1 h, a solution of ZnBr_2_ (1.1 g, 4.8 mmol, 1.5 eq.) was added at − 78 °C. After stirring at room temperature for 30 min, a solution of Pd_2_(dba)_3_ (100 mg, 0.109 mmol, 3.5 mol%), PPh_3_ (173 mg, 0.660 mmol, 21 mol%), and *ortho*-iodobromobenzene (0.40 mL, 3.11 mmol, 1.00 eq.) in THF (3.0 mL) was added. Stirring was maintained overnight. The reaction was quenched by NH_4_Cl aq., and the mixture was extracted into Et_2_O. The organic layer was dried over MgSO_4_ and concentrated under reduced pressure. Purification by column chromatography (silica gel, DCM: *c*-Hex = 1:5) afforded 2-bromo-4′-methoxy-biphenyl **10** (287 mg, 1.09 mmol, 35%) as a white solid. ^1^H NMR (CDCl_3_, 400.13 MHz): δ 7.65 (d, ^3^*J*(H,H) = 8.0 Hz, 1H), 7.36–7.31 (m, 4H), 7.19–7.15 (m, 1H), 6.96 (d, ^3^*J*(H,H) = 8.8 Hz, 1H), 3.86 (s, 3H).

### Synthesis of Bis(4′-methoxybiphenyl-2-yl)silane 1a [27]

 *t*-BuLi (1.7 M in pentane, 1.3 mL, 2.2 mmol, 2.0 eq.) was added to a solution of 2-bromo-4′-methoxybiphenyl **10** (287 mg, 1.09 mmol, 1.0 eq.) in Et_2_O (5.0 mL) at − 78 °C and the mixture was stirred for 1 h at r.t.. The pale brown solution was added to a solution of tetrachlorosilane (0.06 mL, 0.5 mmol, 0.46 eq.) in Et_2_O (0.5 mL) at − 78 °C and stirred for 3 h at r.t.. The resulting pale orange suspension was added to a solution of LiAlH_4_ (1 M in Et_2_O, 2.2 mL, 2.2 mmol, 2.0 eq.) at 0 °C and stirred overnight at r.t.. The orange suspension was quenched with H_2_O and 10% aq. NaOH, filtered through Celite, extracted into Et_2_O and dried over MgSO_4_. After removing solvents, purification by column chromatography (silica gel, *c*-Hex:EtOAc:NEt_3_ = 50:1:0.1) afforded bis(4′-methoxybiphenyl-2-yl)silane **1a** (49 mg, 0.12 mmol, 11%) as a white solid. ^1^H NMR (CDCl_3_, 250 MHz): δ 7.42–7.34 (m, 4H), 7.26–7.17 (m, 4H), 7.08 (d, ^3^*J*(H,H) = 8.8 Hz, 4H), 6.78 (d, ^3^*J*(H,H) = 8.8 Hz, 4H), 4.59 (s, 2H), 3.82 (s, 6H).

### Synthesis of 2,2′-Dimethoxy-9,9′-spiro-9-silabifluorene 2a [27]

 A solution of [Rh(cod)Cl]_2_ (5 mg, 0.01 mmol, 9.8 mol%) and (*R*)-BINAP (16 mg, 0.026 mmol, 25 mol%) in 1,4-dioxane (2.0 mL) was stirred for 30 min at r.t.. A portion of the red mixture (0.1 mL) was transferred to bis(4′-methoxybiphenyl-2-yl)silane **1a** (41 mg, 0.10 mmol, 1.0 eq.) in a separate Schlenk vessel, and heated at 135 °C for 3 h. Afterwards, solvent was removed *in vacuo* and the residue was purified by column chromatography (silica gel, hexane:EtOAc = 50:1) to afford 2,2′-dimethoxy-9,9′-spiro-9-silabifluorene **2a** (33 mg, 0.084 mmol, 84%) as a white solid. ^1^H NMR (CDCl_3_, 250 MHz): δ 7.86–7.81 (m, 4H), 7.47 (td, ^3^*J*(H,H) = 7.8 Hz, ^4^*J*(H,H) = 1.3 Hz, 2H), 7.39 (d, ^3^*J*(H,H) = 6.5 Hz, 2H), 7.15 (t, ^3^*J*(H,H) = 6.8 Hz, 2H), 7.03 (dd, ^3^*J*(H,H) = 8.5 Hz, ^4^*J*(H,H) = 2.8 Hz, 2H), 6.93 (d, ^3^*J*(H,H) = 2.5 Hz, 2H), 3.74 (s, 6H). The *ee* was determined on a Dr. Maish Chiral AM column with *n*-hexane/*iso*-propanol = 95/5, flow = 1 mL/min, oven temp. = 40 °C; 79% *ee*.

### Synthesis of 1-Bromonaphthalen-2yl trifluoromethanesulfonate 11 [30]

 Pyridine (2.8 mL, 35 mmol, 1.2 eq.) was added to a mixture of 1-bromonaphthalen-2-ol (6.7 g, 30 mmol, 1.0 eq.) in dichloromethane (40 mL) at − 10 °C. Triflic anhydride (5.6 mL, 33 mmol, 1.1 eq.) was gradually added to the reaction mixture and stirred for 30 min at − 10 °C. After stirring at r.t. for 2 h, water was added to the mixture at − 10 °C. The mixture was extracted into dichloromethane, the organic layer was washed with 1M HCl and brine, then dried over MgSO_4_ and filtered. Purification by column chromatography (silica gel, *c*-Hex:EtOAc = 50:1) afforded 1-bromonaphthalen-2yl trifluoromethanesulfonate **11** (9.17 g, 0.0258 mmol, 85%) as a white solid. ^1^H NMR (CDCl_3_, 250 MHz): δ 8.32 (d, ^3^*J*(H,H) = 8.3 Hz, 1H), 7.70 (d, ^3^*J*(H,H) = 8.5 Hz, 2H), 7.74–7.59 (m, 2H), 7.44 (d, ^3^*J*(H,H) = 9.0 Hz, 1H).

### Synthesis of 1-Bromo-2-phenylnaphthalene **5** [31]

 A solution of PhMgBr (3 M in Et_2_O, 10 mL, 29 mmol, 1.2 eq.) was added to a mixture of 1-bromonaphthalen-2-yl trifluoromethanesulfonate **11** (8.6 g, 24 mmol, 1.0 eq.), lithium bromide (2.1 g, 13 mmol, 0.54 eq.), Pd_2_(dba)_3_ (334 mg, 0.365 mmol, 1.5 mol%) and 1,3-(diphenylphosphino)propane (301 mg, 0.730 mmol, 3.0 mol%) at 0 °C. After stirring the brown solution overnight at r.t., NH_4_Cl aq. was added at 0 °C. The organic layer was separated and the aqueous layer was extracted into EtOAc. The combined organic layers were dried over MgSO_4_, filtered and concentrated *in vacuo*. Purification by column chromatography (silica gel, *c*-Hex:CH_2_Cl_2_ = 3:1) afforded 1-bromo-2-phenylnaphthalene (5.6 g, 20 mmol, 81%) as a white solid. ^1^H NMR (CDCl_3_, 250 MHz): δ 8.43 (d, ^3^*J*(H,H) = 8.5 Hz, 1H), 7.90–7.84 (m, 2H), 7.68–7.41 (m, 8H).

### Reaction of (2-Phenylnaphthalen-1-yl)lithium with 0.5 Equivalents of SiCl_4_

 *n*-BuLi (1.6 M in hexanes, 1.6 mL, 2.6 mmol, 1.1 eq.) was added to a solution of 1-bromo-2-phenylnaphthalene **5** (700 mg, 2.47 mmol, 1.0 eq.) in Et_2_O (10 mL) at − 78 °C and the mixture was stirred for 1 h at − 78 °C. The pale yellow suspension was added to a solution of tetrachlorosilane (0.14 mL, 1.2 mmol, 0.49 eq.) in Et_2_O (6.0 mL) at − 78 °C and stirred for 3 h at r.t.. The reaction mixture was added to a solution of LiAlH_4_ (1 M in Et_2_O, 2.5 mL, 2.5 mmol, 1.0 eq.) at 0 °C and stirred for 7 h at r.t.. The resulting yellow suspension was quenched with H_2_O and 10% aq. NaOH, filtered through Celite, extracted into Et_2_O, dried over MgSO_4_ and filtered. After removing solvents *in vacuo*, purification by column chromatography (silica gel, *c*-Hex:EtOAc = 40:1) afforded bis(2-phenylnaphthalen-1-yl)silane **3a** (19 mg, 0.044 mmol, 4%) as a white solid and (2-phenylnaphthalen-1-yl)silane **6** (116 mg, 0.410 mmol, 33%) as a colorless oil. Data of bis(2-phenylnaphthalen-1-yl)silane: ^1^H NMR (CDCl_3_, 400.13 MHz): δ 8.00 (d, ^3^*J*(H,H) = 8.5 Hz, 2H), 7.82–7.74 (m, 4H), 7.38 (td, ^3^*J*(H,H) = 7.0 Hz, ^4^*J*(H,H) = 1.0 Hz, 2H), 7.31–7.21 (m, 4H), 7.16–7.08 (m, 10H), 5.19 (s, 2H). ^13^C NMR (CDCl_3_, 126 MHz): δ 145.2 (q), 142.0 (q), 140.4 (q), 140.2 (q), 138.2 (CH), 130.5 (CH), 130.2 (CH), 129.2 (q), 127.9 (CH), 124.7 (CH), 124.6 (CH), 123.9 (CH), 123.2 (CH), 122.3 (CH). HR-MS (FD): calcd for C_32_H_24_Si 436.1647, found 436.1638.

### Reaction of (2-Phenylnaphthalen-1-yl)lithium with 0.9 Equivalents of SiCl_4_

 *t*-BuLi (1.7 M in pentane, 1.2 mL, 2.0 mmol, 2.0 eq.) was added to a solution of 1-bromo-2-phenylnaphthalene **5** (283 mg, 1.00 mmol, 1.0 eq.) in Et_2_O (5.0 mL) at − 78 °C, the mixture was stirred for 1 h at − 78 °C. The pale yellow suspension was added to a solution of tetrachlorosilane (0.10 mL, 0.87 mmol, 0.87 eq.) in Et_2_O (0.5 mL) at − 78 °C and stirred overnight at r.t.. The yellow suspension was added to a solution of LiAlH_4_ (1 M in Et_2_O, 3.0 mL, 3.0 mmol, 3.0 eq.) at 0 °C and stirred for 6 h at r.t.. The mixture was quenched with H_2_O and 10% aq. NaOH, filtered through Celite, extracted with Et_2_O, dried over MgSO_4_ and filtered. After removing solvents *in vacuo*, purification by column chromatography (silica gel, hexane) afforded (2-phenylnaphthalen-1-yl)silane **6** (111 mg, 0.474 mmol, 47%) as a colorless oil. ^1^H NMR (CDCl_3_, 400.13 MHz): δ 8.21 (d, ^3^*J*(H,H) = 8.4 Hz, 2H), 7.96 (d, ^3^*J*(H,H) = 8.4 Hz, 1H), 7.91 (d, ^3^*J*(H,H) = 8.4 Hz, 1H), 7.39–7.62 (m, 8H), 4.20 (s, 3H). ^13^C NMR (CDCl_3_, 126 MHz): δ 150.4 (q), 144.2 (q), 138.1 (q), 132.0 (q), 130.4 (CH), 129.6 (CH), 128.9 (CH), 128.3 (CH), 128.2 (CH), 127.9 (CH), 127.6 (CH), 126.8 (CH), 125.8 (CH), 125.1 (q). GC-MS (LR-EI) *m*/*z* calcd for C_16_H_14_Si 234.1, found 234.1.

### Synthesis of 11,11′-Spirobi[benzo[b]naphtho[2,1-d]silole] **4a**

 The solution of [Rh(cod)Cl]_2_ (2 mg, 0.004 mmol, 17 mol%) and (*R*)-BINAP (10 mg, 0.016 mmol, 70 mol%) in 1,4-dioxane (0.8 mL) was stirred for 30 min at r.t.. A portion of the red mixture (0.03 mL) was transferred to bis(2-phenylnaphthalen-1-yl)silane **3a** (10 mg, 0.023 mmol, 1.0 eq.), and heated at 135 °C for 3 h. After solvents were removed *in vacuo*, purification by column chromatography (silica gel, *c*-Hex:EtOAc = 50:1) afforded 11,11′-spirobi[benzo[*b*]naphtho[2,1-*d*]silole] **4a** as a white solid, which was confirmed by ^1^H NMR measurements. ^1^H NMR (500.23 MHz, CD_2_Cl_2_, 296 K): δ 8.21 (d, ^3^*J*(H,H) = 8.8 Hz, 2H; 3H), 8.13 (d, ^3^*J*(H,H) = 7.6 Hz, 2H; H12), 8.09 (d, ^3^*J*(H,H) = 8.5 Hz, 2H; 4H), 7.81 (d, ^3^*J*(H,H) = 8.2 Hz, 2H; 6H), 7.60 (t, ^3^*J*(H,H) = 7.7 Hz, 2H; 13H), 7.43 (d, ^3^*J*(H,H) = 6.9 Hz, 2H; H15), 7.29‒7.23 (m, 4H; H7 and H14), 7.16 (d, ^3^*J*(H,H) = 8.2 Hz, 2H; H9), 7.06 (t, ^3^*J*(H,H) = 7.6 Hz, 2H; H8); ^13^C{^1^H} NMR (125.78 MHz, CD_2_Cl_2_, 296 K): δ 150.42 (C11), 149.81 (C2), 137.47 (C10), 134.54 (C15), 133.66 (C5), 133.17 (C16), 132.70 (C4), 131.90 (C13), 131.40 (C1), 128.88 (C6), 128.66 (C9), 128.55 (C14), 127.43 (C8), 126.23 (C7), 122.09 (C12), 120.43 (C3); ^1^H-^29^Si-HMBC NMR (400.13, 79.49 MHz, (CD_3_)_2_SO, 296 K): δ − 8.64; HR-MS (EI): calcd for C_32_H_20_Si 432.1334, found 432.1330. Mp 275.7 °C (decomp.). The *ee* was determined on a Chiracel OD-H column with *n*-hexane/*iso*-propanol = 90/10, flow = 0.7 mL/min, oven temp. = 40 °C; 52% ee.

### Synthesis of 3-Bromo-2-phenylbenzo[b]thiophene **12** [32]

 Benzo[b]thiophene (9.6 g, 71.5 mmol, 1.0 eq.) was dissolved in DCM (286 mL) and acetic acid (286 mL), to this solution *N*-bromosuccinimide (31.8 g, 178.8 mmol, 2.5 eq.) was added in small portions over the course of an hour at 0 °C. After 70 min, the ice bath was removed and the mixture was warmed to room temperature. After 9 days of stirring at r.t., the red mixture was quenched with 10% aq. KOH (200 mL), 5% aq. NaHCO_3_ (300 mL) and 5% aq. Na_2_S_2_O_3_ (300 mL). The organic phase was dried over MgSO_4_, filtered and concentrated *in vacuo*. Purification by column chromatography (silica gel, *c*-Hex) afforded **12** as a white solid (15.4 g, 73%). ^1^H NMR (500.23 MHz, (CD_3_)_2_SO, 296 K): δ 8.23 (d, ^3^*J*(H,H) = 8.51 Hz, 1H), 8.19 (d, ^3^*J*(H,H) = 9.14 Hz, 1H), 8.12 (d, ^3^*J*(H,H) = 7.88 Hz, 1H), 7.81 (t, ^3^*J*(H,H) = 7.73 Hz, 1H), 7.73 (t, ^3^*J*(H,H) = 7.57 Hz, 1H), 7.66 (d, ^3^*J*(H,H) = 9.14 Hz, 1H).

### Synthesis of 2-Phenylbenzo[b]thiophene **7c** [33]

 2,3-Dibromobenzo[b]thiophene **12** (4.5 g, 15.3 mmol, 1.0 eq.), phenylboronic acid (1.866 g, 15.3 mmol, 1.0 eq.), Pd(PPh_3_)_4_ (0.354 g, 0.306 mmol, 0.02 eq.) and sodium carbonate (6.657 g, 62.73 mmol, 4.1 eq.) were dissolved in a mixture of degassed 1,4-dioxane (160 mL) and water (32 mL). The yellow solution was refluxed for two nights under nitrogen atmosphere at 110 °C. Solvents were removed *in vacuo* and the residue was dissolved in DCM, washed with brine and the organic phase was dried over MgSO_4_, filtered and concentrated *in vacuo*. Purification by column chromatography (silica gel, *c*-Hex) afforded **7c** a white solid (2.99 g, 67.5%). ^1^H NMR (500.23 MHz, CDCl_3_, 293 K): δ 7.87 (d, ^3^*J*(H,H) = 8.5 Hz, 1H, H4), 7.82 (d, ^3^*J*(H,H) = 8.0 Hz, 1H, H7), 7.76 (d, ^3^*J*(H,H) = 8.0 Hz, 2H, H10), 7.52–7.38 (m, 2H, H5, H6, H11, H12); ^13^C{^1^H} NMR (125.78 MHz, CDCl_3_, 293 K): δ 139.09 (C3), 138.18 (C1), 137.65 (C8), 133.01 (C9), 129.62 (2 CH, C10), 128.76 (CH, C12), 128.56 (2 CH, C11), 125.43 (CH, C6), 125.20 (CH, C5), 123.63 (CH, C4), 122.15 (CH, C7), 104.0 (C7).

### Synthesis Bis(2-phenylbenzo[b]thiophen-3-yl)silane **3c**

 Magnesium (0.28 g, 11.5 mmol, 2.3 eq.) was added to an oven dried reflux setup and dry THF (3 mL) was added. 3-bromo-2-(3,5-dimethylphenyl)benzo[b]thiophene **7c** (1.44 g, 5 mmol, 1 eq.) was dissolved in THF (6 mL) and slowly added to the reflux setup. 1,2-dibromoethane (0.02 mL, 0.23 mmol, 0.046 eq.) was then added and the suspension was refluxed at 66 °C for 2.5 h. Trichlorosilane (0.338 g, 2.5 mmol, 0.5 eq.) was dissolved in dry THF (3 mL) in a separate flame dried Schlenk flask and then added to the yellow solution. The mixture was stirred for 15 min at 60 °C. Next, LiAlH_4_ (1 M in Et_2_O, 1.56 mL, 3.75 mmol, 0.75 eq.) was added dropwise at − 78 °C and the solution was stirred overnight. The blue clear solution was quenched by adding saturated aqueous Rochelle salt dropwise at − 78 °C. The white suspension was extracted using DCM (3x), dried over NaSO_4_, filtered and concentrated *in vacuo*. The product was isolated via column chromatography (silica gel, *c*-Hex:DCM = 100:0 / 90:10 / 50:50) and washed with *n*-pentane to obtain **3c** as a white solid (635.8 mg, 56.8%). ^1^H NMR (500.23 MHz, CDCl_3_, 293 K): δ 7.82 (d, ^3^*J*(H,H) = 7.5 Hz, 2H, H4), 7.64 (d, ^3^*J*(H,H) = 8.0 Hz, 2H, H7) 7.34–7.19 (m, 14H, H5, H6, H10, H11, H12), 5.14 (s, 2H, Si–*H*;); ^13^C{^1^H} NMR (125.78 MHz, CDCl_3_, 293 K): δ 155.0 (C1), 145.1 (C3), 141.0 (C8), 135.2 (C9), 129.8 (C10), 128.5 (C12), 128.1 (C11), 124.7 (C7), 124.5 (CH, C5), 124.2 (CH, C7), 123.5 (C2), 121.8 (C4). ^29^Si-HMBC NMR (400.13, 79.49 MHz, CDCl_3_, 296 K): δ − 62.3 (Si). HR-MS (FD): calcd for C_28_H_20_S_2_Si 448.0776, found 448.0788.

### Synthesis of 10,10′-Spirobi[benzo[b]benzo [4,5] silolo[2,3-d]thiophene] Using (R)-BINAP **4c**

 A solution of [Rh(cod)Cl]_2_ (2.5 mg, 0.0051 mmol, 5.1 mol%) and (*R*)-BINAP (10 mg, 0.016 mmol, 16 mol%) in 1,4-dioxane (1.0 mL) was stirred for 1 h at r.t.. A portion of the red mixture (0.1 mL) was transferred to bis(2-phenylbenzo[*b*]thiophen-3-yl)silane **3c** (45 mg, 0.10 mmol, 1.0 eq.), and heated at 135 °C for 3 h. After solvents were removed *in vacuo*, purification by column chromatography (silica gel, hexane:EtOAC = 50:1) afforded spirosilane **4c** (4 mg, 0.008 mmol, 8.8%) after washing with *n*-pentane as a white solid, which was confirmed by ^1^H NMR measurements. ^1^H NMR (500.23 MHz, THF-*d*_8_, 294 K): δ = 7.89 (d, ^3^*J*(H,H) = 8.0 Hz, 2H; 7H), 7.68 (d, ^3^*J*(H,H) = 7.5 Hz, 2H; H14), 7.52 (t, ^3^*J*(H,H) = 7.5 Hz, 2H, H13), 7.39 (d, ^3^*J*(H,H) = 7.0 Hz, 2H; H11), 7.25–7.21 (m, 6H; H4, H6, H12), 7.11 (t, ^3^*J*(H,H) = 7.5 Hz, 2H; H5); ^13^C{^1^H} NMR (125.78 MHz, CD_2_Cl_2_, 296 K): δ 161.26 (C1), 145.76 (C9), 143.46 (C8), 141.55 (C3), 134.23 (C11), 133.57 (C10), 131.93 (C13), 129.81 (C2), 128.58 (C12), 125.36 (C5), 124.77 (C6), 124.66 (C4), 123.38 (C7), 122.43 (C14); ^1^H-^29^Si-HMBC NMR (400.13, 79.49 MHz, THF-*d*_8_, 296 K): δ -24.1 (Si); HR-MS (EI): calcd for C_28_H_16_S_2_Si 444.0463, found 444.0457 ; Mp 215.5 °C (decomp.).The *ee* was determined on a Dr. Maisch chiral AM column with *n*-hexane/*iso*-propanol = 95/5, flow = 0.7 mL/min, oven temp. = 40 °C; 54% *ee*.

### Synthesis of 10,10′-Spirobi[benzo[b]benzo [4,5] silolo[2,3-d]thiophene] Using (R)-SEGPHOS **4c**

A solution of [Rh(cod)Cl]_2_ (2.5 mg, 0.0051 mmol, 5.1 mol%) and (*R*)-SEGPHOS (7.3 mg, 0.012 mmol, 12 mol%) in 1,4-dioxane (1.0 mL) was stirred for 30 min at r.t.. A portion of the red mixture (0.1 mL) was transferred to bis(2-phenylbenzo[*b*]thiophen-3-yl)silane **3c** (45 mg, 0.10 mmol, 1.0 eq.) and heated at 135 °C for 3 h. After all volatiles were removed *in vacuo*, purification by column chromatography (silica gel, hexane:EtOAc = 50:1) afforded the desired spirosilane (10.8 mg, 0.024 mmol, 24.0%) as a white solid, which was confirmed by ^1^H NMR measurements. ^1^H NMR (CDCl_3_, 400.13 MHz): δ 7.90 (d, ^3^*J*(H,H) = 8.0 Hz, 2H), 7.68 (d, ^3^*J*(H,H) = 7.6 Hz, 2H), 7.52 (td, ^3^*J*(H,H) = 7.6 Hz, ^4^*J*(H,H) = 0.8 Hz, 2H), 7.42 (d, ^3^*J*(H,H) = 7.2 Hz, 2H), 7.29 (d, ^3^*J*(H,H) = 8.8 Hz, 2H), 7.26–7.22 (m, 4H), 7.14 (td, ^3^*J*(H,H) = 8.0 Hz, ^4^*J*(H,H) = 0.8 Hz, 2H). The *ee* was determined on a Chiracel OD-H column with *n*-hexane/*iso*-propanol = 90/10, flow = 0.7 mL/min, oven temp. = 40 °C; 84% *ee*.

### Synthesis of 10,10′-Spirobi[benzo[b]benzo [4,5] silolo[2,3-d]thiophene] Using (R)-DTBM-SEGPHOS **4c**

A solution of [Rh(cod)Cl]_2_ (2.5 mg, 0.0051 mmol, 5.1 mol%) and (*R*)-DTBM-SEGPHOS (14 mg, 0.012 mmol, 12 mol%) in 1,4-dioxane (1.0 mL) was stirred for 30 min at r.t.. A portion of the red mixture (0.1 mL) was transferred to bis(2-phenylbenzo[*b*]thiophen-3-yl)silane **3c** (45 mg, 0.10 mmol, 1.0 eq.) and heated at 135 °C for 3 h. After all volatiles were removed *in vacuo*, purification by column chromatography (silica gel, hexane:EtOAc = 50:1) afforded the desired spirosilane (14 mg, 0.031 mmol, 31.1%) as a white solid, which was confirmed by ^1^H NMR measurements. ^1^H NMR (CDCl_3_, 400.13 MHz): δ 7.90 (d, ^3^*J*(H,H) = 8.0 Hz, 2H), 7.68 (d, ^3^*J*(H,H) = 7.6 Hz, 2H), 7.52 (td, ^3^*J*(H,H) = 7.6 Hz, ^4^*J*(H,H) = 0.8 Hz, 2H), 7.42 (d, ^3^*J*(H,H) = 7.2 Hz, 2H), 7.29 (d, ^3^*J*(H,H) = 8.8 Hz, 2H), 7.26–7.22 (m, 4H), 7.14 (td, ^3^*J*(H,H) = 8.0 Hz, ^4^*J*(H,H) = 0.8 Hz, 2H). The *ee* was determined on a Chiracel OD-H column with *n*-hexane/*iso*-propanol = 90/10, flow = 0.7 mL/min, oven temp. = 40 °C; 84% *ee*.

### Synthesis of 10,10′-Spirobi[benzo[b]benzo [4,5] silolo[2,3-d]thiophene] Using (R)-(+)-MeO-BIPHEP **4c**

A solution of [Rh(cod)Cl]_2_ (2.5 mg, 0.0051 mmol, 5.1 mol%) and (*R*)-(+)-MeO-BIPHEP (14 mg, 0.024 mmol, 24 mol%) in 1,4-dioxane (1.0 mL) was stirred for 30 min at r.t.. A portion of the red mixture (0.1 mL) was transferred to bis(2-phenylbenzo[*b*]thiophen-3-yl)silane **3c** (45 mg, 0.10 mmol, 1.0 eq.) and heated at 135 °C for 3 h. After solvents were removed *in vacuo*, purification by column chromatography (silica gel, hexane:EtOAc = 50:1) afforded the desired spirosilane (33 mg, 0.073 mmol, 73.3%) as a white solid, which was confirmed by ^1^H NMR measurements. ^1^H NMR (CDCl_3_, 400.13 MHz): δ 7.90 (d, ^3^*J*(H,H) = 8.0 Hz, 2H), 7.68 (d, ^3^*J*(H,H) = 7.6 Hz, 2H), 7.52 (td, ^3^*J*(H,H) = 7.6 Hz, ^4^*J*(H,H) = 0.8 Hz, 2H), 7.42 (d, ^3^*J*(H,H) = 7.2 Hz, 2H), 7.29 (d, ^3^*J*(H,H) = 8.8 Hz, 2H), 7.26–7.22 (m, 4H), 7.14 (td, ^3^*J*(H,H) = 8.0 Hz, ^4^*J*(H,H) = 0.8 Hz, 2H). The *ee* was determined on a Chiracel OD-H column with *n*-hexane/*iso*-propanol = 90/10, flow = 0.7 mL/min, oven temp. = 40 °C; 45% *ee*.

### Synthesis of 10,10′-Spirobi[benzo[b]benzo [4,5] silolo[2,3-d]thiophene] Using (R)-3,5-tBu-4-MeO-MeOBIPHEP **4c**

A solution of [Rh(cod)Cl]_2_ (2.5 mg, 0.0051 mmol, 5.1 mol%) and (*R*)-3,5-*t*Bu-4-MeO-MeOBIPHEP (14 mg, 0.012 mmol, 12 mol%) in 1,4-dioxane (1.0 mL) was stirred for 30 min at r.t.. A portion of the red mixture (0.1 mL) was transferred to bis(2-phenylbenzo[*b*]thiophen-3-yl)silane **3c** (45 mg, 0.10 mmol, 1.0 eq.) and heated at 135 °C for 3 h. After solvents were removed *in vacuo*, purification by column chromatography (silica gel, *c*-Hex:EtOAc = 50:1) afforded the desired spirosilane (26 mg, 0.058 mmol, 57.8%) as a white solid, which was confirmed by ^1^H NMR measurements. ^1^H NMR (CDCl_3_, 400.13 MHz): δ 7.90 (d, ^3^*J*(H,H) = 8.0 Hz, 2H), 7.68 (d, ^3^*J*(H,H) = 7.6 Hz, 2H), 7.52 (td, ^3^*J*(H,H) = 7.6 Hz, ^4^*J*(H,H) = 0.8 Hz, 2H), 7.42 (d, ^3^*J*(H,H) = 7.2 Hz, 2H), 7.29 (d, ^3^*J*(H,H) = 8.8 Hz, 2H), 7.26–7.22 (m, 4H), 7.14 (td, ^3^*J*(H,H) = 8.0 Hz, ^4^*J*(H,H) = 0.8 Hz, 2H). The *ee* was determined on a Chiracel OD-H column with *n*-hexane/*iso*-propanol = 90/10, flow = 0.7 mL/min, oven temp. = 40 °C; 13% *ee*.

### Synthesis of 2-(2-Bromophenyl)benzo[b]thiophene **8**

 *n*-BuLi (1.6 M in hexanes, 7.0 mL, 11.0 mmol, 1.1 eq.) was added to a solution of 2-phenylbenzo[*b*]thiophene (1.4 g, 10 mmol, 1.0 eq.) in THF (12 mL) at − 78 °C. After stirring for 1 h at − 78 °C, a solution of ZnBr_2_ (2.7 g, 12 mmol, 1.2 eq.) in THF (8.0 mL) was added to the pale pink suspension at − 78 °C. After stirring the white suspension at r.t. for 30 min, a solution of Pd_2_(dba)_3_ (230 mg, 0.251 mmol, 2.5 mol%), PPh_3_ (430 mg, 1.64 mmol, 16 mol%), and *ortho*-iodobromobenzene (1.3 mL, 10 mmol, 1.0 eq.) in THF (9.0 mL) was added at r.t. and the mixture was stirred overnight. The reaction was quenched using aq. NH_4_Cl, extracted into Et_2_O, dried over MgSO_4_, filtered and concentrated *in vacuo*. Purification by column chromatography (silica gel, 1st column; hexane:CH_2_Cl_2_ = 10:1, 2nd column; hexane) afforded 2-(2-bromophenyl)benzo[*b*]thiophene **8** (1.4 g, 4.8 mmol, 48%) as a white solid. ^1^H NMR (CDCl_3_, 250 MHz): δ 7.89–7.80 (m, 2H), 7.71 (d, ^3^*J*(H,H) = 8.0 Hz, 1H), 7.56 (dd, ^3^*J*(H,H) = 7.5 Hz, ^3^*J*(H,H) = 1.5 Hz, 1H), 7.32–7.42 (m, 3H), 7.50 (s, 1H), 7.22 (dd, ^3^*J*(H,H) = 7.5 Hz, ^4^*J*(H,H) = 1.8 Hz, 1H). ^13^C NMR (CDCl_3_, 126 MHz): δ 140.4 (q), 140.0 (q), 139.9 (q), 135.4 (q), 133.8 (CH), 132.3 (CH), 129.7 (CH), 127.5 (CH), 124.6 (CH), 124.0 (CH), 123.1 (q), 122.2 (CH). GC-MS *m*/*z* calcd. for C_14_H_9_BrS 288.0 (^79^Br), found 287.9 (^79^Br).

### Synthesis of Bis(2-(benzo[b]thiophen-2-yl)phenyl)silane **3e**

 *n*-BuLi (1.6 M in hexanes, 2.3 mL, 3.6 mmol, 1.0 eq.) was added to a solution of 2-(2-bromophenyl)benzo[*b*]thiophene **8** (1.0 g, 3.5 mmol, 1.0 eq.) in Et_2_O (10 mL) at − 78 °C and the mixture was stirred for 1 h at r.t.. Tetrachlorosilane (0.2 mL, 1.7 mmol, 0.49 eq.) was added to the yellow solution at − 78 °C and stirred for 21.5 h at r.t.. Afterwards, LiAlH_4_ (1 M in Et_2_O, 3.5 mL, 3.5 mmol, 1.0 eq.) was added at 0 °C and stirred for 5 h at r.t.. The brown suspension was quenched with H_2_O and 10% aq. NaOH, filtered through Celite and volatiles were removed *in vacuo*. Purification by column chromatography (silica gel, hexane) afforded bis(2-(benzo[*b*]thiophen-2-yl)phenyl)silane **3e** (120 mg, 0.267 mmol, 8%) as a white solid, which still contains minor impurities that could not be separated. The product was used without further purification. ^1^H NMR (CDCl_3_, 400.13 MHz): δ 7.74 (d, ^3^*J*(H,H) = 8.0 Hz, 2H), 7.64 (d, ^3^*J*(H,H) = 7.2 Hz, 2H), 7.45 (d, ^3^*J*(H,H) = 7.6 Hz, 2H), 7.46–7.28 (m, 8H) 7.13 (t, ^3^*J*(H,H) = 7.0 Hz, 2H), 6.99 (s, 2H), 4.87 (s, 2H). GC-MS *m*/*z* calcd for C_28_H_20_S_2_Si 448.1, found 446.1.

### Synthesis of (2-(Benzo[b]thiophen-2-yl)phenyl)silane **9**

 *n*-BuLi (1.6 M in hexanes, 0.70 mL, 1.1 mmol, 1.1 eq.) was added to a solution of 2-(2-bromophenyl)benzo-[*b*]thiophene **8** (289 g, 1.00 mmol, 1.0 eq.) in Et_2_O (3.0 mL) at − 78 °C, the mixture was stirred for 30 min at − 78 °C and for 30 min at r.t.. A solution of tetrachlorosilane (0.06 mL, 0.5 mmol, 0.5 eq.) in Et_2_O (0.50 mL) was added to the yellow solution at 0 °C and stirred for 3 h at r.t.. Next, LiAlH_4_ (1 M in Et_2_O, 1.0 mL, 1.0 mmol, 1.0 eq.) was added to the yellow suspension at − 78 °C and stirred overnight at r.t.. The mixture was quenched with water and 10% aq. NaOH, filtered through Celite and the solvent was removed *in vacuo*. Purification by column chromatography (silica gel, *c*-Hex) afforded (2-(benzo[*b*]thiophen-2-yl)phenyl)silane **9** (30 mg, 0.13 mmol, 13%) as a colorless oil. ^1^H NMR (CDCl_3_, 400.13 MHz): δ 7.86 (d, ^3^*J*(H,H) = 7.6 Hz, 1H), 7.82 (d, ^3^*J*(H,H) = 7.6 Hz, 1H), 7.77 (d, ^3^*J*(H,H) = 7.2 Hz, 1H), 7.57 (d, ^3^*J*(H,H) = 7.6 Hz, 1H), 7.50 (t, ^3^*J*(H,H) = 7.6 Hz, 1H), 7.41–7.32 (m, 4H), 4.22 (s, 3H). GC-MS *m*/*z* calcd for C_14_H_12_SSi 240.0, found 240.0.

### Synthesis of 3-Bromo-2-(3,5-dimethylphenyl)benzo[b]thiophene **7d**

 2,3-Dibromobenzo[b]thiophene (4.0 g, 13.6 mmol, 1.0 eq.), (3,5-dimethylphenyl)boronic acid (2.04 g, 13.6 mmol, 1.0 eq.), Pd(PPh_3_)_4_ (0.31 g, 0.272 mmol, 0.02 eq.) and sodium carbonate (5.91 g, 55.76 mmol, 4.1 eq.) were dissolved in a mixture 1,4-dioxane (75 mL) and water (75 mL), after which the mixture was degassed. After reflux overnight at 115 °C, the yellow mixture was quenched with 2M HCl and filtered with Celite using 1,4-dioxane and concentrated *in vacuo*. The yellow mixture was extracted into EtOAc, dried over MgSO_4_, filtered and concentrated *in vacuo*. **7d** was isolated as a white powder using column chromatography (*c*-Hex) (2.33 g, 7.3 mmol, 54%). ^1^H NMR (500.23 MHz, CDCl_3_, 293 K): δ 7.87 (d, ^3^*J*(H,H) = 8.0 Hz, 1H, H4), 7.81 (d, ^3^*J*(H,H) = 8.0 Hz, 1H, H7), 7.48 (t, ^3^*J*(H,H) = 7.4 Hz, 1H, H5), 7.42–7.38 (m, 3H, H6, H10) 7.08 (s, 1H, 12H), 2.41 (s, 6H, 13H); ^13^C{^1^H} NMR (125.78 MHz, CDCl_3_, 293 K): δ 139.3 (C3), 138.7 (C1), 138.3 (C9), 137.8 (C8), 133.0 (C11), 130.7 (CH, C12), 127.5 (C13), 125.5 (CH, C6), 125.3 (CH, C5), 123.7 (CH, C4), 122.3 (CH, C7), 104.8 (C2), 21.5 (C13).

### Synthesis of Bis(2-(3,5-dimethylphenyl)benzo[b]thiophen-3-yl)silane **3d**

 Magnesium (0.28 g, 11.5 mmol, 2.3 eq.) dispersed in dry THF (3 mL) was added to an oven dried setup. 3-bromo-2-(3,5-dimethylphenyl)benzo-[b]thiophene **7d** (1.58 g, 5 mmol, 1.0 eq.) was dissolved in THF (6 mL) and slowly added to the reflux setup followed by addition of 1,2-dibromoethane (0.02 mL, 0.23 mmol, 0.046 eq.) and the mixture was refluxed at 66 °C for 2.5 h. Trichlorosilane (0.338 g, 2.5 mmol, 0.5 eq.) was dissolved in dry THF (3 mL) in a separate flame dried Schlenk vessel, added to the yellow solution and the resulting mixture was stirred for 15 min at 60 °C. Next, LiAlH_4_ (1 M in Et_2_O, 1.56 mL, 3.75 mmol, 0.75 eq.) was added dropwise at − 78 °C and the solution was stirred overnight. The reaction was quenched by dropwise adding a saturated aqueous Rochelle salt at − 78 °C. It was extracted into DCM (3x) and dried over NaSO_4_, filtered and concentrated *in vacuo*. The product isolated via column chromatography (*c*-Hex) was not the desired product, but 2-(3,5-dimethylphenyl)benzo[b]thiophene was obtained instead (106.9 mg, 21%). ^1^H NMR (400.13 MHz, CDCl_3_, 293 K): δ 7.82 (d, ^3^*J*(H,H) = 7.8 Hz, 1H), 7.70–7.69 (m, 2H), 7.26–7.19 (m, 4H), 6.88 (s, 1H), 2.20 (s, 1H). ^1^H-^29^Si-HMBC NMR (400.13, 79.49 MHz, CDCl_3_, 295 K): δ − 62.51 (*Si*–H).
